# Evaluating Oculomotor Tests before and after Vestibular Rehabilitation in Patients with Parkinson's Disease: A Pilot Pre-Post Study

**DOI:** 10.1155/2022/6913691

**Published:** 2022-02-28

**Authors:** Amirabas Abasi, Reza Hoseinabadi, Parvin Raji, Joseph H. Friedman, Mohammad-Reza Hadian

**Affiliations:** ^1^Department of Occupational Therapy, School of Rehabilitation, Tehran University of Medical Sciences, Tehran, Iran; ^2^Department of Audiology, School of Rehabilitation, Tehran University of Medical Sciences, Tehran, Iran; ^3^Department of Neurology, Warren Alpert School of Medicine at Brown University, Providence, RI, USA; ^4^School of Rehabilitation, Brain and Spinal Injury Research Center (BASIR), Institute of Neuroscience, Tehran University of Medical Sciences (TUMS), International Campus TUMS, Tehran, Iran

## Abstract

**Introduction:**

The elderly population is commonly affected by balance and gait disorders that increase the risk of falls. Pivotal systems for efficient postural control are sensory, motor, visual, vestibular, and cognitive. Disruption in any of these systems could lead to postural instability. Vestibular rehabilitation is a set of exercises that positively affect the primary components of the central sensory-motor integration, including somatosensory, visual, and vestibular systems. Accordingly, we hypothesized that vestibular rehabilitation exercises might improve both oculomotor functions and upright postural control in patients with Parkinson's disease.

**Materials and Methods:**

11 idiopathic Parkinson's patients voluntarily participated in this study based on inclusion criteria: central vestibular dysfunction and the Hoehn and Yahr scale scores less than or equal to 3. Videonystagmography (VNG) and the Berg Balance Scale (BBS) scores were measured at the baseline. Then, the patients underwent vestibular rehabilitation training for 24 sessions (3 sessions per week). The VNG and BBS were measured again after 48 hours of the completion of the last session of the training.

**Result:**

After completing vestibular rehabilitation sessions, there were significant improvements in balance (*P* ≤ 0.001). Eye-tracking and gaze function statistically improved in 7 patients and 6 patients, respectively.

**Conclusion:**

Vestibular rehabilitation produced positive effects on oculomotor function and balance in a small cohort of people with PD. Consequently, it could be considered as a possible effective intervention for Parkinson's patients. This trial is registered with IRCT201709123551N6.

## 1. Introduction

Parkinson's disease (PD) is a neurodegenerative disorder defined by tremors, rigidity, bradykinesia, and postural instability (PI). Nonmotor symptoms affect the vast majority of patients, with autonomic dysfunction, cognitive abnormalities, dementia, sleep disorders, anosmia, and pain [1–3]. Postural instability negatively affects gait and balance and therefore can increase the risk of falls and is a significant cause of morbidity and mortality in PD [4].

PI in patients with PD is the reduced ability to preserve balance due to a decrease in demonstrating postural control strategies [2], specifically the adoption of a flexed posture and trunk rotation [5]. Dopaminergic neuronal deficits, comorbid white matter disease, and degeneration of the cholinergic system are thought to be the main causes of motor impairments in PD patients [6]. Postural instability in these patients often leads to falls, which comprise about 75% of hospitalizations [7, 8]. A meta-analysis in this area of research reported that falls occur in about half of the PD patients during a short period of only 3 months [9]. Nevertheless, PI among PD patients is poorly responsive to current drug or nondrug interventions [10]. As the disease progresses, PI worsens and often leads to more vulnerable consequences [7, 10–17]. Therefore, early intervention and constant monitoring in decreasing and preventing the economical and emotional burden of PI in PD patients are essential.

Regaining and retraining postural control and stability are critical for PD patients. Postural stability is the ability to maintain equilibrium during standing or walking; through that, a perfect movement will occur from the very first phase of initiation, prediction, and reaction to perturbations; accordingly, taking steps and efficient postural control strategies (i.e., ankle, hip, and stepping) will be achieved [18]. Therefore, having good postural control requires the integration of diverse areas in the brain such as sensory, motor, visual, vestibular, and cognitive circuits. Disruption in any of these circuits may lead to vulnerable consequences such as falling [4].

In previous studies, oculomotor abnormalities were demonstrated in Parkinson's patients [19, 20]. The major abnormalities involve decreased saccade velocity, increased saccade latency, and hypometric saccades [21]. These abnormalities contribute to visual processing deficits that may impair the usual visual compensatory mechanisms to correct or modify imbalance. Additionally, the oculomotor abnormalities in PD are referred to as impairment of their pathways in the midbrain, cerebellum, and basal ganglia that are interconnected with sensory-motor parts of the brain that are responsible for postural control [22, 23].

Sensory-motor parts of the brain including vestibular and somatosensory systems are the primary components of central sensory-motor integration which are improved by vestibular rehabilitation. Vestibular rehabilitation is a comprehensive approach for patients with postural control disorders which is thought to work by improving postural reactions during standing and walking through compensatory and adaptation mechanisms. It is accomplished by retraining similar exercises that stimulate specific areas of the brain that are associated with equilibrium and sensory-motor circuits such as the frontal lobe and other cortical structures [23].

Despite a diligent search, we found no reports on the effect of vestibular rehabilitation on oculomotor function in patients with PD. It was suggested that vestibular rehabilitation in patients with vestibular hypofunction improved vestibulo-ocular reflex gain during active head rotation, independent of peripheral vestibulo-ocular reflex gain recovery. Accordingly, the risk of falling remarkably diminished, and no falls were reported among the intervention group after three weeks of oculomotor and gaze stability exercises [24]. Vestibular rehabilitation also had positive effects on self-reported dizziness and balance in patients with Parkinson's disease [23, 25]. The main aim of this pilot study was to investigate the effects of vestibular rehabilitation on oculomotor functions. We hypothesized that eye movements, which were evaluated with videonystagmography (VNG), might improve after vestibular rehabilitation exercises in patients with PD.

## 2. Method

In this pilot study, eleven PD patients diagnosed by a senior neurologist voluntarily participated based on the following inclusion criteria: the Hoehn and Yahr scale scores less than or equal to 3; from stage I without any balance impairment; according to the senior neurologist's opinion, subjects whose BBS scores were 45 assigned as H&Y II; stage III bilateral or midline involvement; or mild to moderate disability with impaired postural reflexes and physically independent. Central vestibular dysfunction in participants was detected by a senior audiologist. Central vestibular dysfunction was defined as a lesion within the central nervous system structures including the brain, cerebellum, or brain stem. Some manifestations of these dysfunctions in VNG include pure vertical nystagmus and lack of nystagmus suppression in caloric tests. Additionally, patients with a score lower than 45 (independent in doing all items by providing some excessive endeavor to regain the balance) on the Berg Balance Scale (BBS) were enrolled to identify patients with balance impairment. The exclusion criteria were as follows: a severe osteoarticular disease with an inability to perform the proposed exercises, or previous performance of oculomotor or gaze stability exercises. Blindness in one eye, or other eye abnormalities, BPPV, or other vestibular problems that may not have shown up on vestibular testing were detected and excluded under the supervision of a senior neurologist [21, 25].

### 2.1. Standard Protocol Approvals

Qualified and satisfied patients were accepted after signing an informed consent form, and the study was approved by the Iranian Registry Center of the Clinical Trials and the Ethical Committee of Tehran University of Medical Sciences (IR.TUMS.FNM.REC.1396.3210).

### 2.2. Pre-Post Assessments

After providing informed written consent, the patients underwent a comprehensive physical and neurologic assessment. Outcome measures were assessed twice: (1) before the vestibular rehabilitation started and (2) after 48 hours when they completed the 24 sessions (3 sessions per week) of training.

### 2.3. Videonystagmography Test

Evaluation of eye movements started with saccades. Saccades are the ability to maintain an image at the center of the visual line and are defined as the fastest eye movements in humans. Accuracy, velocity, and latency are parameters that are primarily important in this oculomotor test. Saccades were considered abnormal when their velocity of them was lower than 430°/sec and/or their velocity was more than 250 ms. Hypometrics and hypermetric saccades were considered abnormal, too. In addition to cortical activity, diverse parts of the brain are involved in controlling saccade velocity, latency, and accuracies, such as vestibulo-cerebellar connections and pontine reticular formation [26].

In contrast to saccades, smooth pursuit eye movements are defined as the ability to maintain gaze on moving objects in the visual field. In the smooth pursuit test, gains must be between 80 and 120, and the phase should be less than ±10. Moreover, the presence of corrective saccades was considered as a sign of abnormal smooth pursuit. The presence of any nystagmus in the gaze test was considered as an abnormal finding. Smooth pursuit abnormalities are due to abnormalities in the central vestibular dysfunction [26].

In this study, an American device House Infrared/Video ENG System was utilized by a senior audiologist to provide oculomotor tests [26]. A sample test's result is attached in Appendix B.

Also, a senior audiologist supervised and performed videonystagmography (VNG) tests to assess the central vestibular function in participants; it was advised 48 hours before the test to discontinue consuming caffeine (coffee, tea, and cola) before going to sleep. Additionally, all medications were pleased to be halted, except emergency medications that are prescribed by their physician to be necessary [21].

The Berg Balance scale (BBS) has 16 items and a total score ranging from 0 to 56 (0 = dependent and 56 = the best balance). A score less than 46 indicates a significant risk of falling. The Berg Balance Scale is used in most balance studies and has strong evidence of value in identifying balance disorders [27].

### 2.4. Interventions

The vestibular rehabilitation exercises that lasted for about 60 minutes in every 24 sessions were performed in three categories included the following: standing, walking, and oculomotor training. Exercises while standing on a firm surface, foam, a trampoline, and a balance board. Subjects were asked to follow orders including maintaining their eyes closed and open; turning their heads to the sides, upward, and downward; and alterations in the center of gravity were also provided by throwing and catching a ball. Oculomotor exercises included tracking a marker in diverse directions (each took about 1 minute), gazing at the marker and rotating the head from side to side, and looking at two markers as fast as possible. All these training sessions took about 10 minutes of the whole treatment session and are explained in detail in Appendix A [23, 28, 29].

## 3. Statistical Analyses

Descriptive statistics are reported as mean ± SD. The paired *t-*test was used to compare the pre- and postmeasurements. All statistical analyses were performed by a senior biostatistician. A *P* value of 0.05 or less was considered statistically significant.

## 4. Results

Demographics and inclusion criteria are listed in Table 1. As can be seen, 11 subjects participated (mean age 65.16, with 7 subjects in H&Y stage III and 4 in stage II). There was a significant difference in terms of the Berg Balance Scale (*P* ≤ 0.001) after vestibular rehabilitation sessions (Table 2). In Table 3, the oculomotor test results for each patient are provided separately. In the tracking section of the oculomotor test, 9 patients were in the abnormal range at baseline, 7 of whom returned to the normal range after training sessions. Likewise, in the saccade section, in which the two items velocity and latency of the eye movement in the horizontal direction were assessed: 10 patients were abnormal in both items at baseline, 5 of whom became normal in velocity and 1 of whom returned to normal in both velocity and latency. In the gaze section of the oculomotor test, 5 patients had horizontal nystagmus on upward gaze at baseline, while after vestibular rehabilitation sessions, 1 became normal. In the gaze section, the severity of nystagmus in 2 patients had decreased.

## 5. Discussion

This study demonstrated that twenty-four sessions of vestibular rehabilitation resulted in significant improvement in eye-tracking movements. The velocity of saccadic eye movements improved in 6 patients; however, there was no improvement in the latency of saccadic eye movements. We have not found any reports on the effect of vestibular rehabilitation on the oculomotor function of Parkinson's patients. Therefore, from a neurophysiological perspective, we do not know how vestibular rehabilitation affected eye movement improvement. Only one study reported that vestibular rehabilitation enhanced visual and vestibular inputs in the Sensorial Organization Test in Parkinson's patients [23]. This test is designed to separately evaluate somatosensory, visual, and vestibular inputs in diverse conditions in patients transitioning from stable to unstable surfaces [23].

We demonstrated a remarkable improvement in the Berg Balance Scale. This is in line with what Hadian et al. reported that vestibular rehabilitation protocol, in which oculomotor training was thought to play a key role in the coordination of head and trunk movement, can significantly improve balance scores [25]. Following this, some authors suggested that head and trunk coordination is of great importance since this is crucial for regaining equilibrium and neuromuscular reorganization in PD patients [4, 19, 23, 30]. It is possible that oculomotor training included in vestibular rehabilitation, which improved VNG results in the eye-tracking test and the velocity of saccadic eye movements of half of the participants, had a positive impact on the coordination of their limbs during static balance. Further studies are required to explicitly evaluate the effects of oculomotor training on both static and dynamic balance in PD.

This pilot study either helps support the previous observations or expands on them. Previous studies have reported impaired eye movements and balance in Parkinson's patients. Accordingly, it has been suggested that there are correlations between eye movement impairments, gait, balance, sleep disturbance, and cognitive impairments in Parkinson's patients [31]. Nevertheless, there is not sufficient evidence to conclude the effects of oculomotor training on balance in PD. However, in poststroke rehabilitation, some studies reported that oculomotor training and gaze stability exercises had improved the risk of falls [32, 33].

It is worth mentioning that, although we did not exclusively evaluate the effects of oculomotor training on both static and dynamic balance, we found improvement in some eye movements that magnify the importance of taking deeper steps toward understanding the effects of tailored interventions on eye movements' abnormalities in PD patients.

Vestibular rehabilitation is a combination of exercises that include postural control and ocular motor exercises, as mentioned. So, patients may have improved their ocular motor deficits because they have trained. As previous studies reported that oculomotor pathology in PD is indicated in pathways that are highly interconnected with postural control, more studies are required to understand if any changes could be observed in the level of brain connections in larger PD patients [22, 23].

This study has several limitations: one of them is the small size of the population and its lack of reflection of the PD community as a whole. The lack of follow-up kept us from assessing the single most important potential outcome variable, a reduction in falls. Although there are many questions in this field of research requiring further investigations, this study provided insight for future studies with larger and more diverse sample sizes and more outcome measures such as the Time UP and GO test and other functional assessment tools.

## 6. Conclusion

Our results, if confirmed, but other studies are required, suggest that vestibular rehabilitation is an underutilized approach to improve balance as well as other problems in patients with a variety of neurological disorders. It seems that vestibular rehabilitation can be performed in a wide array of clinical settings as a comprehensive, reliable, safe, and available training in Parkinson's patients.

## Figures and Tables

**Table 1:** Demographic data of the patients (*n* = 11).

Variable	PD patients
Age (years)	65.16 (8.05)
Male/female	4/7
Years with a diagnose	3.33 (1.55)
H&Y scale II/III	4/7
BMI	24.30 (3.18)

The number in parentheses is the standard deviation.

**Table 2:** Measures of pre- and postvestibular rehabilitation.

Variable	Before	After	Changes	95% CI	*P*
Berg balance scale	43.27 (2.72)	50.09 (2.30)	6.81 (3.09)	4.74 to 8.89	0.001

The number in parentheses is the standard deviation.

**Table 3:** Videonystagmography outcomes pre- and postvestibular rehabilitation.

Variable	Tracking		Saccade		Gaze	
	Pre	Post	Pre	Post	Pre	Post
*P*1	Abnormal	Abnormal	V: abnormal L: abnormal	V: normal L: abnormal	Normal	Normal
*P*2	Normal	Normal^a^	V: abnormal L: abnormal	V: normal L: abnormal	Horizontal nystagmus in upward	Normal
*P*3	Abnormal	Normal	V: abnormal L: abnormal	V: normal L: abnormal	Horizontal nystagmus in upward	Horizontal nystagmus in upward
*P*4	Abnormal	Normal	V: abnormal L: abnormal	V: abnormal L: abnormal	Normal	Normal
*P*5	Abnormal	Normal	V: abnormal L: abnormal	V: normal L: abnormal	Normal	Normal
*P*6	Abnormal	Normal	V: abnormal L: abnormal	V: normal L: normal	Horizontal nystagmus in upward	Normal
*P*7	Abnormal	Normal	V: abnormal L: abnormal	V: abnormal L: abnormal	Horizontal nystagmus in upward	Horizontal nystagmus in upward (severity decreased from 3˚ to 1°)
*P*8	Abnormal	Normal	V: normal L: abnormal	V: normal L: abnormal	Normal	Horizontal nystagmus in upward
*P*9	Abnormal	Normal	V: abnormal L: abnormal	V: abnormal L: abnormal	Normal	Normal
*P*10	Normal	Normal^a^	V: abnormal L: abnormal	V: abnormal L: abnormal	Normal	Horizontal nystagmus in upward
*P*11	Abnormal	Abnormal	V: abnormal L: abnormal	V: abnormal L: abnormal	Horizontal nystagmus in upward	Horizontal nystagmus in upward (severity decreased)

V = velocity; L = latency; a = being in a safe normal range after training sessions.

## Data Availability

The data that support the findings of this study are included within the article.

## Apendix

### A. Vestibular Rehabilitation

Data can be found online at https://doi.org/10.2522/ptj.20100399.

### B. A Sample of the VNG Test Result of a Participant

1. Before vestibular rehabilitation sessions.

2. After vestibular rehabilitation sessions.
